# Leukocyte Immunoglobulin-Like Receptors A2 and A6 are Expressed in Avian Macrophages and Modulate Cytokine Production by Activating Multiple Signaling Pathways

**DOI:** 10.3390/ijms19092710

**Published:** 2018-09-11

**Authors:** Anh Duc Truong, Deivendran Rengaraj, Yeojin Hong, Ha Thi Thanh Tran, Hoang Vu Dang, Viet Khong Nguyen, Hyun S. Lillehoj, Yeong Ho Hong

**Affiliations:** 1 Department of Animal Science and Technology, Chung-Ang University, Anseong 17546, Korea; truonganhduc84@gmail.com (A.D.T.); deivnedran@snu.ac.kr (D.R.); lovejin5873@naver.com (Y.H.); 2 Department of Biochemistry and Immunology, National Institute of Veterinary Research, 86 Truong Chinh, Dong Da, Hanoi 100000, Vietnam; thanhhavty@yahoo.com (H.T.T.T.); dangnivr@yahoo.com (H.V.D.); nguyenvietkhong@yahoo.com (V.K.N.); 3 Animal Biosciences and Biotechnology Laboratory, Agricultural Research Services, United States Department of Agriculture, Beltsville, MD 20705, USA; Hyun.Lillehoj@ARS.USDA.GOV

**Keywords:** chicken, LILRA, cytokine, MHC class I, signaling pathway

## Abstract

The activating leukocyte immunoglobulin-like receptors (LILRAs) play an important role in innate immunity. However, most of the LILRA members have not been characterized in avian species including chickens. The present study is the first attempt at cloning, structural analysis and functional characterization of two LILRAs (LILRA2 and LILRA6) in chickens. Multiple sequence alignments and construction of a phylogenetic tree of chicken LILRA2 and LILRA6 with mammalian proteins revealed high conservation between chicken LILRA2 and LILRA6 and a close relationship between the chicken and mammalian proteins. The mRNA expression of *LILRA2* and *LILRA6* was high in chicken HD11 macrophages and the small intestine compared to that in several other tissues and cells tested. To examine the function of LILRA2 and LILRA6 in chicken immunity, *LILRA2* and *LILRA6* were transfected into HD11 cells. Our findings indicated that LILRA2 and LILRA6 are associated with the phosphorylation of Src kinases and SHP2, which play a regulatory role in immune functions. Moreover, LILRA6 associated with and activated MHC class I, β2-microglobulin and induced the expression of transporters associated with antigen processing but LILRA2 did not. Furthermore, both LILRA2 and LILRA6 activated JAK-STAT, NF-κB, PI3K/AKT and ERK1/2 MAPK signaling pathways and induced Th1-, Th2- and Th17-type cytokines and Toll-like receptors. Collectively, this study indicates that LILRA2 and LILRA6 are essential for macrophage-mediated immune responses and they have the potential to complement the innate and adaptive immune system against pathogens.

## 1. Introduction

The leukocyte immunoglobulin-like receptor (LILR) family consists of members that play inhibitory or activating roles on the genes of the proteins involved with the immune system and expressed in several primary innate immune cells (monocyte/macrophages, dendritic cells and CD4^+^/CD8^+^ T cells) [1,2,3] and cell lines (HEK293 T cells, MDCK cells, HL-60 cells, thymoma BWZ.36 cells, epithelial cells and T cells) [1,4,5,6]. The LILR genes are highly homologous in the sequence of the extracellular regions and different in their sequences of the intracellular regions [7,8]. The gene structure of the activating LILRs (LILRAs) commonly encode a signal peptide, two or four immunoglobulin (Ig)-like domains, a transmembrane domain and a cytoplasmic tail that associates with the Fc receptor γ (FcRγ) chain containing immunoreceptor tyrosine-based activation motifs (ITAMs) to control innate and adaptive immune responses [9]. Based on the interaction with human leukocyte antigen (HLA) class I molecules, human LILRAs are categorized into LILRA group 1 (LILRA1–3) and LILRA group 2 (LILRA4–6) members [7,10]. Moreover, LILRAs have been demonstrated to play important roles in infection or autoimmune diseases such as HIV infection [11], multiple sclerosis [12], atopic dermatitis [13] and rheumatoid arthritis [14]. It has been reported that LILRA1, 3, 4 and 5 bind with HLA-G, HLA-C and classical HLAs [3,7,11,13,14] and regulate adaptive or innate immune pathways such as the ERK/MEK [15], TLR [3] and JNK/p38MAPK [16] signaling pathways. Additionally, they have been reported to upregulate the cytokines IL-1R, IL-4, IL-6, IL-10, IL-12, IL-17, TNFα and IFN-γ [3,7,11,14]. 

In humans, LILRA2 is a type of innate immune receptor in the host immune system that plays a role in the immune response to microbial pathogens such as *Mycoplasma hyorhinis*, *Streptococcus pneumonia*, *Candida albicans* and *Legionella pneumophila* [1] and conditions such as inflammatory bowel disease [17] and rheumatoid arthritis [18]. In addition, human LILRA6 is correlated with susceptibility to atopic dermatitis [13]. Cross-linking of LILRA2 and LILRA6 on the surface of macrophages induces and regulates cytokines such as IL-4, IL-10, IL-17, TNFα and IFN-γ [3,6,9]. This suggests that LILRA2 and LILRA6 play a role in the modulation of immune responses but the fundamental mechanisms by which LILRA2 and LILRA6 regulate cytokine production are not well characterized in mammalian species. Currently, no data exist regarding the role of LILRA2 and LILRA6 in the activation of immune signaling pathways in mammalian and avian species, although it was recently discovered that LILRA2 and LILRA6 interact with SHP2 and LILRA6 binds with an MHC class I ligand but not with LILRA2 in dendritic cells (DC), suggesting that they could shape immune responses in monocytes [1,7].

Recent studied demonstrated that chicken leukocyte immunoglobulin receptor (LIR) are shown highly homologous with chicken Ig-like receptors (CHIR) family genes and also play an important role to recognize avian influenza [4]. On the other hand, some members of the Ig superfamily in chicken were identified and characterized of functions that may be involved in immune responses such as triggering receptor expressed on myeloid cells (TREM), cluster of differentiation (CD) 300, signal-regulatory protein alpha (SIRP), CHIR-A, CHIR-B and CHIR-AB homologs [4,19,20,21]. Moreover, there is no information on the primary structure and function of LILRA2 and LILRA6 in avian species. Therefore, we cloned the entire open reading frame of *LILRA2* and *LILRA6* to characterize avian LILRA2 and LILRA6 using chickens as an avian model organism. In this study, we also demonstrate the expression and functional analysis of chicken *LILRA2* and *LILRA6* in the chicken macrophage (HD11) cell line. The findings of this study indicate that *LILRA2* and *LILRA6* associated with MHC class I, non-classical β2-microglobulin (β2m), the phosphorylation of Src kinases and SHP2 and activates the JAK-STAT, NF-κβ, PI3K/AKT and ERK1/2 MAPK signaling pathways and modulates cytokine production.

## 2. Results 

### 2.1. Cloning and Identification of Chicken LILRA2 and LILRA6

**Figure 1:**
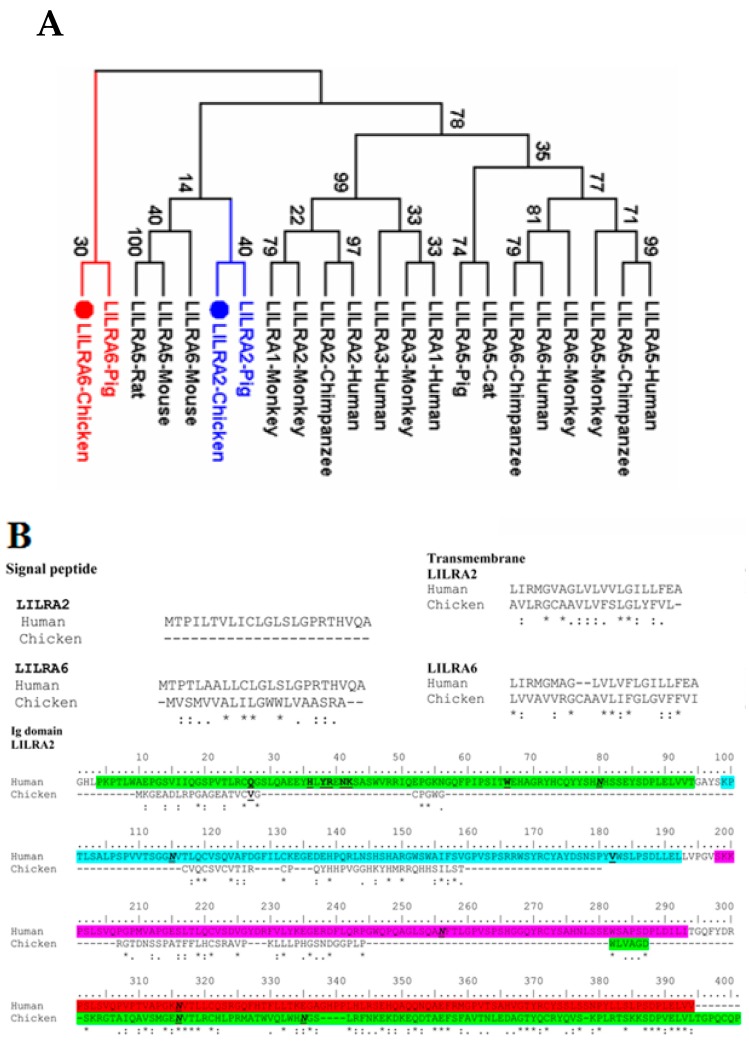

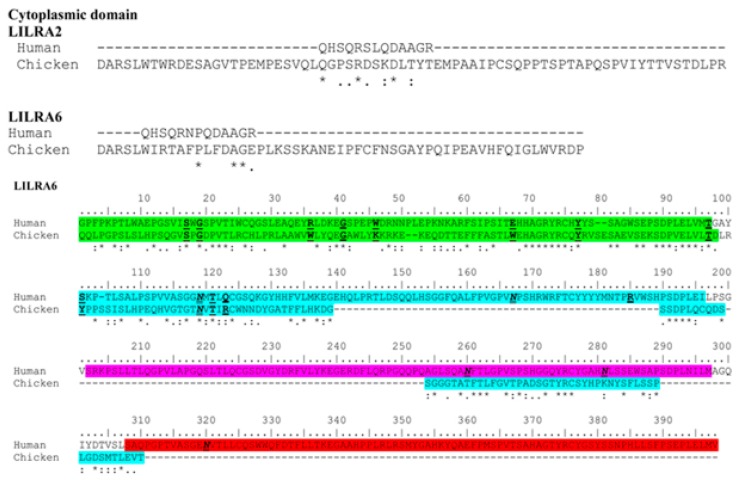
Phylogenetic and sequence alignment analysis of chicken LILRA2 and LILRA6. (**A**) Phylogenetic tree indicating the relationships of chicken LILRA2 and LILRA6 with mammalian LILRA2 and LILRA6; (**B**) Multiple sequence alignment of the predicted chicken LILRA2 and LILRA6 with human LILRA2 and LILRA6. Asterisks (*) indicate identical amino acid residues. Single dots (.) indicate homologous amino acid changes and double dots (:) indicate conserved amino acid changes. N-linked glycosylation sites and binding site are indicated in italic-underline and bold-underline, respectively. Four Ig domains (D1–D4 domains) are indicated in green, turquoise, pink and red color, respectively.

The identity and similarity of LILRA2 and LILRA6 between chickens and homologue proteins of other species were analyzed using the Sequence Identity and Similarity (SIAS) program based on the amino acid sequences (Table 1). To clarify the evolutionary relationships of chicken LILRA2 and LILRA6 to those of other species, phylogenetic analysis and sequence alignment were also conducted using the MEGA6 program (Figure 1). Comparison of amino acid identities and similarities of chicken LILRA2 and LILRA6 with mammalian species such as human, monkey, chimpanzee and pig showed 19.70% to 55.37% and 17.81% to 59.78%, respectively (Table 1). The identity and similarity between the predicted chicken LILRA2 and LILRA6 amino acid sequences is 50.08% and 66.13%, respectively (Table 1). The results of phylogenetic analyses showed that chicken LILRA2 and LILRA6 are closely related to those of mammalian species. Particularly, these chicken receptors were more closely related to homologous receptors of pig than those of other species (Figure 1A). 

The secondary structure of human LILRA2 and LILRA6 includes a signal peptide, four Ig domains (D1–D4 domains) and transmembrane and cytoplasmic domains (Figure 1B). Conceptual translation of the open reading frame showed that the chicken LILRA6 protein consists of 306 amino acids, including a 20-aa signal peptide, 208-aa Ig-like domain (D1–D2 domain), 23-aa transmembrane domain and 55-aa cytoplasmic domain (Figure 1B). Comparison of chicken LILRA6 to the human homologue found that there are two pairs of highly conserved cysteine residues, which form two disulfide bonds (C^26^–C^75^ and C^124^–C^276^) at almost same sites among the LILRA6 D1–D2 domains with known crystal structures for human LILRA6 (Figure 1B). These disulfide bonds are the main structural pattern of the C2 type Ig-like domain in humans and mice [22]. In contrast to LILRA6, chicken LILRA2 (consisting of 320 amino acids) is composed of a 229-aa Ig-like domain (D1 domain) with 1 disulfide bond (C^321^–C^372^), 20-aa transmembrane domain and 71-aa cytoplasmic domain without a signal peptide (Figure 1B). 

Two Ig domains (D1–D2) of group 1 of LILRs in human are arranged in a V-shaped conformation and each domain is composed primarily of β strands arranged into two anti-parallel β sheets and also D1–D2 domain is mainly bind to MHC class I and β2m [10,23]. The positions (R^36^, W^46^, K^41^, D^39^, K^42^ and E^184^, I^100^, Q^18^ and I^92^) of D1–D2 region in group 1 of LILRs binds to MHC class I and β2m that are present in D1–D2 region of human LILRA2 as H^36^, Y^38^, D^41^, K^42^ and E^198^ and V^182^, S^98^, Q^18^ and W^66^, which are not bind to MHC class I and β2m (Figure 1B) [10,23]. In contrast, there are many differences in the corresponding residues between chicken LILRA2 and group 1 of LILRs in human D1–D2 region, explaining LILRA2 in chicken and human likely do not bind to MHC molecules. On the other hand, human LILRA6 (group 2 of LILRs) amino acids associated with MHC class I and β2m ligand binding (S^17^, G^19^, Q/R^36^, G^41^, L/W^46^, Q/E^67^, Y^77^, T^97^, S^101^, T^117^, R/Q^120^, N/T^160^, M/T^178^ and Q/W/R^182^) in Ig domain D1–D2, in which the Ig D1 region (S^17^, G^19^, Q/R^36^, G^41^, L/W^46^, Q/E^67^, Y^77^ and T^97^) strongly bind to MHC class I and β2m [5,10,24] that are highly conserved in chicken and human LILRA6. The results indicated that Ig D1 region of LILRA6 is strongly binds to MHC class I but LILRA2 does not bind to MHC class I and β2m.

Moreover, there are two N-linked glycosylation sites (N^116^ and N^135^) in the D1 domain and one N-linked glycosylation site (N^137^) in the D2 domain of the chicken LILRAs (Figure 1B). The cytoplasmic domains of human and mouse LILRA2 and LILRA6 are short (13-aa) compared to homologous chicken proteins (55- and 71-aa, respectively). The transmembrane regions of chicken LILRA2 and LILRA6 contain an arginine in the 4th and 7th positions (3^rd^ position in human and mouse homologues) at the transition between the transmembrane and cytoplasmic regions, respectively. This sequence pattern is conserved among several activating Ig-like receptors and associated with ITAM-containing Fc epsilon receptor type Iγ (FcɛRIγ) or heterodimers of FcɛRIγ and cluster of differentiation zeta subunit (CD3ζ), which itself is critical for the expression on the cell surface, for delivery of the activation signal pathway and induction of genes expression [9,25]. Therefore, the results indicated that chicken LILRA2 and LILRA6 genes might differ in their capacity of their ligand binding sites and regulate immune responses.

### 2.2. Distribution of LILRA2 and LILRA6 Transcripts in Normal Tissues and Cells 

**Figure 2:**
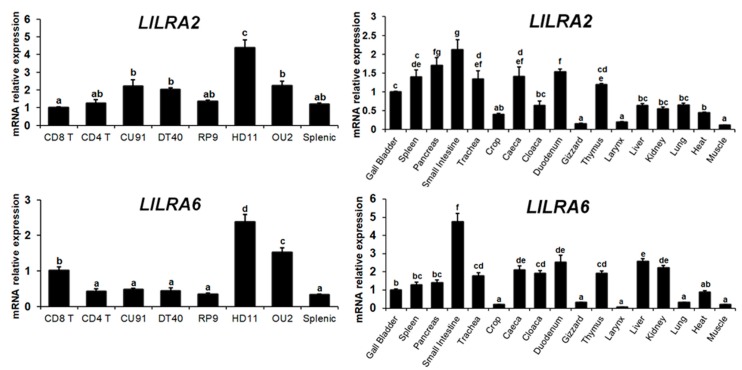
Expression of chicken *LILRA2* and *LILRA6* mRNA in chicken tissues and cells. (**Left**) The expression of *LILRA2* and *LILRA6* mRNA in chicken cells; (**Right**) Expression of *LILRA2* and *LILRA6* mRNA in normal tissues. Gene expression levels were normalized to *GAPDH* and calibrated with CD8 T-cell and gall bladder expression in chicken cell and tissues, respectively. Data are presented as the mean ± SEM of three independent experiments and values with different superscript characters (a, b, c, d, e, f and g) indicate significant differences between the control and treatment groups as determined by one-way ANOVA (*p* < 0.05).

qRT-PCR analysis was used to examine the expression of *LILRA2* and *LILRA6* transcripts in 17 normal tissues and eight types of chicken cells (Figure 2). Expression of *LILRA2* and *LILRA6* transcripts was detected variably in all normal tissues and chicken cells. Comparatively, *LILRA2* mRNA was significantly expressed in HD11, OU2, CU91 and DT40 cells and tissues of the small intestine, pancreas, thymus, spleen, trachea, caeca and duodenum. Moreover, *LILRA6* mRNA showed high expression in HD11 and OU2 cells and tissue of the small intestine. Conversely, crop, gizzard, larynx and muscle tissues weakly expressed *LILRA2*. Low-level *LILRA6* mRNA expression was also observed in crop, gizzard, larynx and muscle tissues. The results of this study indicated that *LILRA2* and *LILRA6* expression was higher in the small intestine and HD11 cells compared to that of other tissues and cells.

### 2.3. Chicken LILRA2 and LILRA6 Binding with MHC Class I 

To confirm the efficiency of eGFP-linked LILRA2, LILRA6 and the mock control to bind to MHC class I, they were transfected into HD11 cells. After transfection, we determined the transfection efficiency in LILRA2 and LILRA6-transfected HD11 cells by EVOS FLoid Cell Imaging microscopy (Figure S1A) and FACS analysis (Figure S1B). The results of microscopy and FACS analysis showed that the transfection efficiency was >77%. Subsequently, eGFP vector, linked LILRA2 and LILRA6 protein expression were determined by western blotting. As shown in Figure S1C, a single band was detected by anti-eGFP mAb in eGFP, eGFP-linked LILRA2 and LILRA6-transfected HD11 cells. Taken together, these data demonstrated that LILRA2 and LILRA6 proteins were constitutively expressed in HD11 cells transfected with our eGFP vector. Moreover, the results of cytotoxicity analysis showed that the proliferation of chicken HD11 cells after transfection was not significantly inhibited or enhanced (Figure S1D, above) but NO production was higher in LILRA2- and LILRA6-eGFP transfected cells than in the control group (Figure S1D, bottom). These results suggest that the chicken LILRA2 and LILRA6 induced the production of reactive oxygen species in the form of NO.

**Figure 3:**
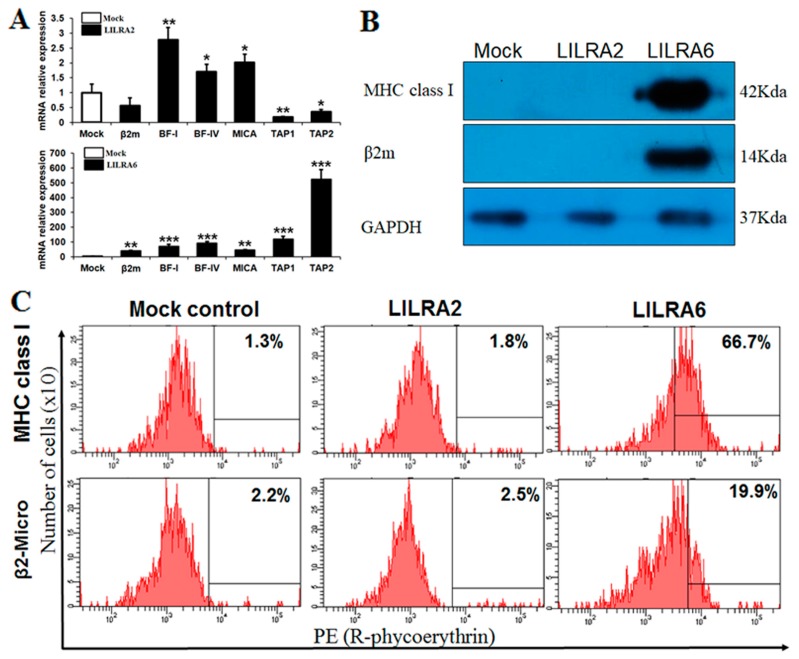
Chicken LILRA2 and LILRA6 binding with MHC class I and β2m. (**A**) qRT-PCR for MHC class I family and related genes in LILRA2 and LILRA6 transfected HD11 cells; (**B**) Western blot for LILRA2 and LILRA6 transfected HD11 cells immunoprecipitated with the MHC class I, β2m mAb, GAPDH antibody and then separated by SDS-PAGE and immunoblotted with secondary antibody; (**C**) FACS analysis of LILRA2 and LILRA6 binding with MHC class I and β2m mAb in HD11 cells. Data are presented as the mean ± SEM of three independent experiments: * *p* < 0.05, ** *p* < 0.01 and *** *p* < 0.001.

In humans, it has been well demonstrated that the LILRA family of proteins bind with MHC class I and β2m [23]. In this study, we transfected eGFP alone or eGFP-linked LILRA2 and LILRA6 into HD11 cells and performed qRT-PCR to examine chicken LILRA binding or upregulation of MHC class I family genes (*MICA*, *BF-I* and *BF-IV*) as well as other genes involved in class I antigen presentation and processing, such as *β2m*, transporter associated with antigen processing 1 (*TAP1*) and *TAP2* (Figure 3A). The results indicated that upregulation of MHC class I family genes and related genes by LILRA6 activation was greater than that by LILRA2. Specifically, *β2m*, *BF-I*, *BF-IV*, *MICA*, *TAP1* and *TAP2* mRNA were upregulated by 39.95-, 70.03-, 90.21-, 44.01-, 118.05- and 522.75-fold, respectively, after LILRA6 activation (Figure 3A). In contrast, *BF-I*, *BF-IV* and *MICA* mRNA were upregulated <3-fold after LILRA2 activation (Figure 3A). Immunoprecipitation analysis was performed to determine whether LILRA2 and LILRA6 proteins bind with MHC class I and β2m (Figure 3B). We found that LILRA6 immunoprecipitates bound to each anti-MHC class I and β2m antibodies. However, LILRA2 was not bound to MHC class I or β2m, suggesting that MHC class I and β2m associate with LILRA6 but not LILRA2 in a chicken macrophage cell line (Figure 3B). Furthermore, FACS analysis using MHC class I and β2m specific antibodies confirmed an increase in MHC class I surface expression in cells transfected with LILRA6 only (Figure 3C). Specifically, LILRA6 was associated with MHC-I and β2m by 66.7% and 19.9%, respectively. Whereas, the LILRA2 was associated with MHC-I and β2m by 1.8% and 2.5%, respectively, which is similar to that of the mock control (Figure 3C). Previous studies have revealed that inhibitory and activating LILRs interact with self MHC class I in *cis, trans* and constitutively induces self-regulatory signaling and it would be the cross-linking of inhibitory and activating receptors, which would have a positive feedback on their expression [26,27]. Our results suggest that MHC class I and related genes can also act as a molecule capable of signaling transcriptional and phenotypical changes in HD11 cells, by itself. Collectively, these results indicate that LILRA6 actively induces the expression of MHC class I and related genes involved in MHC class I antigen presentation and binds with MHC class I and β2m.

### 2.4. Chicken LILRA2 and LILRA6 are Associated with Phosphorylation of Src and SHP2

**Figure 4:**
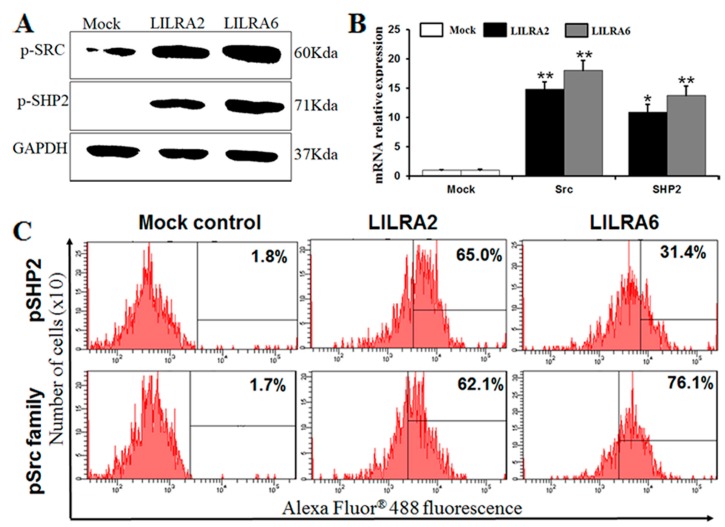
Chicken LILRA2 and LILRA6 are associated with phosphorylation of Src family and SHP2. (**A**) Western blot analysis for phosphorylated Src family members and SHP2 activated by LILRA2 and LILRA6 transfected in HD11 cells; (**B**) qRT-PCR analysis for *Src1* and *SHP2* mRNA transcripts activated by LILRA2 and LILRA6 in HD11 cells; (**C**) FACS analysis for phosphorylated Src family members and SHP2 expressing cells activated by LILRA2 and LILRA6 in HD11 cells. Data are presented as the mean ± SEM of three independent experiments: * *p* < 0.05 and ** *p* < 0.01.

To determine whether chicken LILRA2 and LILRA6 associate with members of the Src family and SHP2, we examined the tyrosine phosphorylation profile after LILRA2- and LILRA6-eGFP transfection in HD11 cells (Figure 4). First, we observed the phosphorylation level of Src family members (Tyr^41^) and SHP2 (Tyr^542^) in response to LILRA2 and LILRA6 transfected HD11 cells by western blotting (Figure 4A). As shown in Figure 4A, LILRA2 and LILRA6 are associated with Src and SHP2 but the levels of signaling proteins were higher in LILRA6 transfected cells. This result suggests that LILRA2 and LILRA6 could associate with Src kinase and SHP2 signaling pathways and may regulate the immune system. Furthermore, the expression levels of Src1 and SHP2 mRNA transcripts were abundantly upregulated in LILRA2 and LILRA6 transfected HD11 cells and slightly higher in LILRA6 activation (Figure 4B). FACS analysis using a Src family (Tyr^41^) and SHP2 (Tyr^542^) antibodies was performed to confirm the expression of Src family members and SHP2 in transfected cells with LILRA2 and LILRA6. The expression levels of Src family members and SHP2 were 62.1% and 65.0% in LILRA2 and 76.1% and 31.4% in LILRA6 transfected cells, respectively (Figure 4C). The expression of SHP2 in western blot, qRT-PCR does not correlate well with flow cytometry data (Figure 4) it may be caused by the interaction between protein-protein in the living cell. Taken together, chicken LILRA2 and LILRA6 could associate with tyrosine phosphorylated Src kinases, SHP2 and play an important role in modulation of signaling pathways in a chicken macrophage cell line.

### 2.5. Chicken LILRA2 and LILRA6 Activate the JAK-STAT Signaling Pathway 

**Figure 5:**
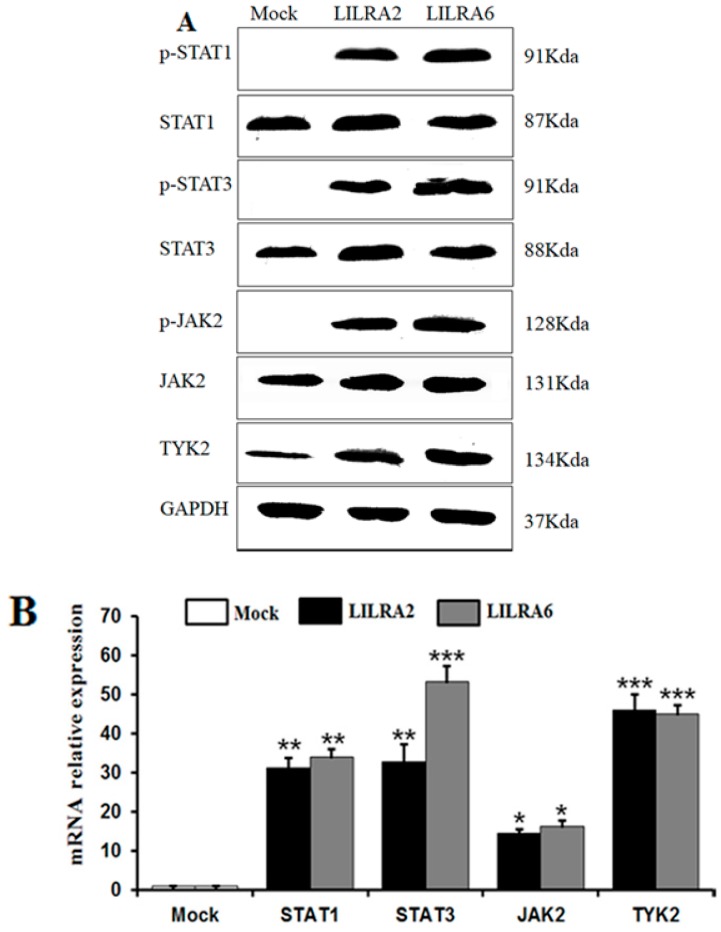

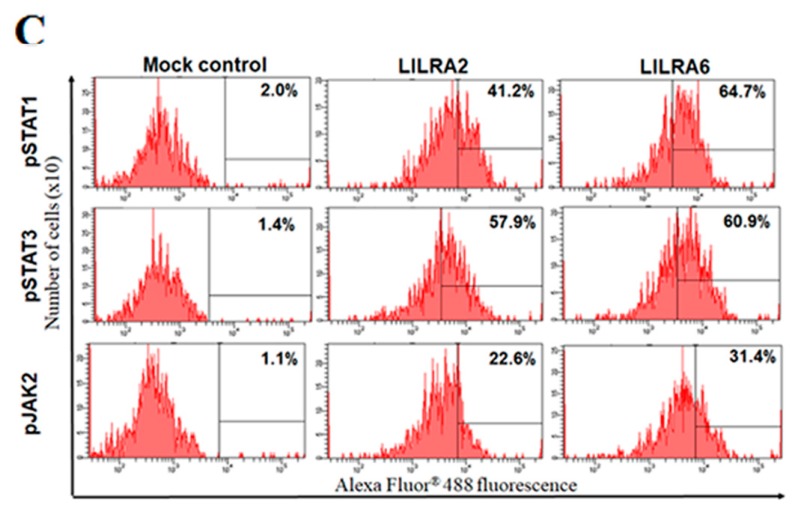
Chicken LILRA2 and LILRA6 regulate the JAK-STAT signaling pathway. (**A**) Western blot of phosphorylated and un-phosphorylated STAT1, STAT3, JAK2 and TYK2 in LILRA2 and LILRA6 transfected HD11 cells; (**B**) qRT-PCR expression of STAT1, STAT3, JAK2 and TYK2 in LILRA2 and LILRA6 transfected HD11 cells; (**C**) FACS analysis of phosphorylated STAT1, STAT3 and JAK2 in LILRA2 and LILRA6 transfected HD11 cells. Data are presented as the mean ± SEM of three independent experiments: * *p* < 0.05, ** *p* < 0.01 and *** *p* < 0.001.

To investigate whether chicken LILRA2 and LILRA6 affect JAK-STAT signaling in HD11 cells, western blotting, FACS analysis and qRT-PCR were performed (Figure 5). Initially, we observed the expression of phosphorylated STAT1 (p-Ser^727^), STAT3 (p-Ser^727^) and JAK2 (p-Tyr^1007^/Tyr^1008^) and un-phosphorylated STAT1/3, JAK2 and TYK2 by western blot analysis (Figure 5A) and FACS (Figure 5C) in HD11 cells transfected with LILRA2 and LILRA6. It was observed that the expression levels of phosphorylated STAT1 (p-Ser^727^), STAT3 (p-Ser^727^) and JAK2 (p-Tyr^1007^/Tyr^1008^) were markedly increased by LILRA2 and LILRA6 activation. Interestingly, the expression levels of signaling proteins were much higher in LILRA6 transfected cells than that in LILRA2 transfected cells (Figure 5A–C). In qRT-PCR analysis, the expression levels of *STAT1*, *STAT3*, *JAK2* and *TYK2* mRNA were significantly upregulated in LILRA2 (31.32-, 34.1-, 14.60- and 16.35-fold, respectively) and LILRA6 (32.71-, 53.21-, 46.08- and 45.0-fold, respectively) transfected HD11 cells (Figure 5B). These results strongly imply that chicken LILRA2 and LILRA6 might act on the JAK-STAT signaling pathway and in turn regulate immune system responses.

### 2.6. Chicken LILRA2 and LILRA6 Activate NF-κB and MAPK Signaling Pathways

**Figure 6:**
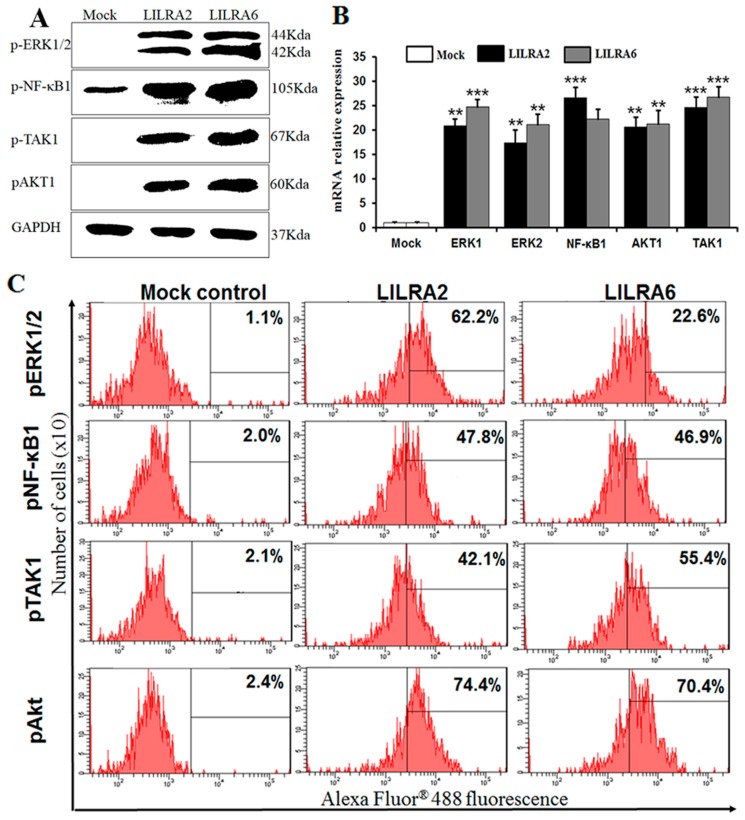
NF-κB and MAPK signaling pathways regulated by LILRA2 and LILRA6. Western blot (**A**) and FACS (**C**) analyses for phosphorylated ERK1/2, NF-κB1, TAK1 and AKT1 in LILRA2 and LILRA6 transfected HD11 cells; (**B**) qRT-PCR expression of ERK1, ERK2, NF-κB1, TAK1 and AKT1 in LILRA2 and LILRA6 transfected HD11 cells. Data are presented as the mean ± SEM of three independent experiments: ** *p* < 0.01 and *** *p* < 0.001.

To determine whether NF-κB, PI3K/AKT and Ras/MAPK signaling pathways are activated by LILRA2 and LILRA6 in HD11 cells, expression levels of phosphorylated NF-κB1 (Ser^933^), TAK1 (Ser^192^), AKT1 (Ser^473^) and p44/42 MAPK (ERK1/2) (Thr^202^/Tyr^204^) were evaluated by western blotting, FACS and qRT-PCR analyses (Figure 6). Phosphorylated ERK1/2, NF-κB1, TAK1 and AKT1 were dramatically increased after LILRA2 and LILRA6 transfection in HD11 cells as detected with phospho-specific antibodies by western blotting (Figure 6A) and FACS analysis (Figure 6C). Specifically, FACS analysis showed that the expression levels of phosphorylated ERK1/2, NF-κB1 and AKT1were higher in LILRA2 transfected cells, while phosphorylated TAK1 was highly expressed in LILRA6 transfected cells. The qRT-PCR results indicated that *ERK1, ERK2, NF-κB1, Akt1* and *TAK1* were significantly upregulated in LILRA2 (20.90-, 17.36-, 26.64-, 20.63- and 24.70-fold, respectively) and LILRA6 (24.75-, 21.16-, 22.30-, 21.34- and 26.80-fold, respectively) transfected HD11 cells (Figure 6B). These results clearly indicate that LILRA2 and LILRA6 regulate the expression and subcellular localization of NF-κB and MAPK signaling molecules in chicken macrophages.

### 2.7. Chicken LILRA2 and LILRA6 Induce Cytokine Production

**Figure 7:**
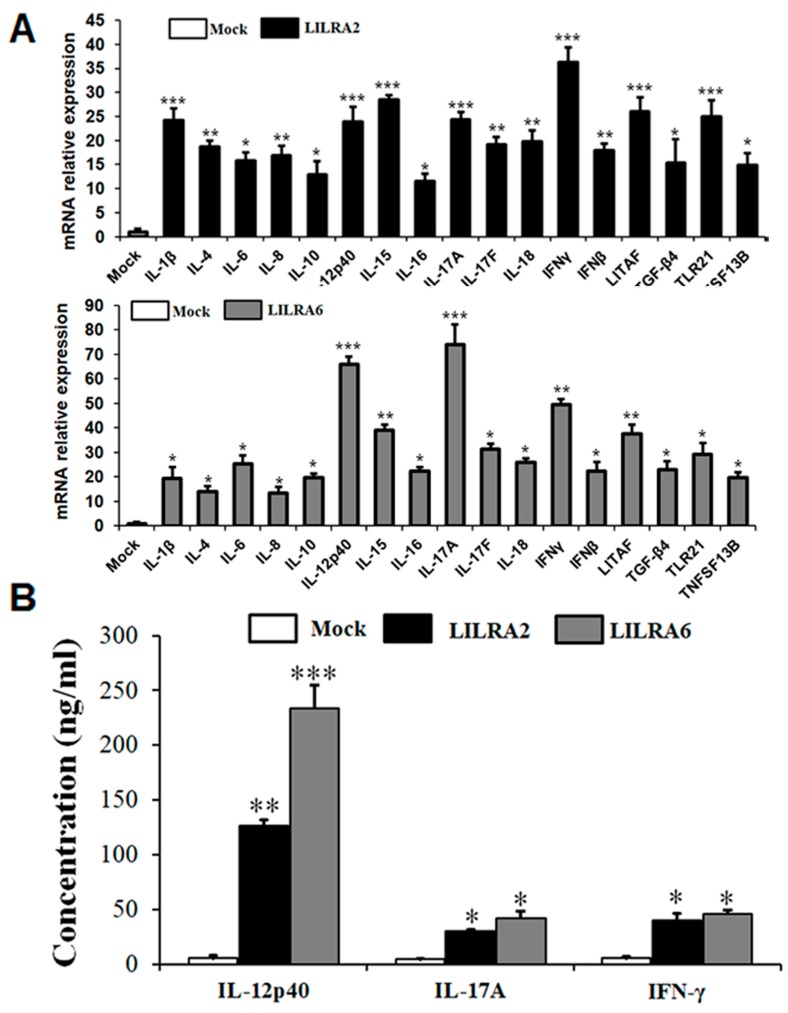
Chicken LILRA2 and LILRA6 activate cytokine production. Cytokine mRNA (**A**) and protein (**B**) expression levels in LILRA2 and LILRA6 transfected HD11 cells. Data are presented as the mean ± SEM of three independent experiments: * *p* < 0.05, ** *p* < 0.01 and *** *p* < 0.001.

To determine the effects of LILRA2 and LILRA6 on cytokines induced in HD11 cells, cells were transfected with LILRA2 and LILRA6 for 72 h. Cell supernatants and extracts of treated and mock control group were collected and analyzed to measure the mRNA and protein expression for several cytokines (Figure 7A). The mRNA expression levels of proinflammatory cytokines (*IFN-β*, *IFN-γ*, *IL-1β*, *IL-6*, *IL-8*, *IL12-p40*, *IL-15*, *IL-16*, *IL-17A*, *IL-17F* and *IL-18*) and anti-inflammatory cytokines (*IL-4* and *IL-10*) were significantly increased in LILRA2 and LILRA6 transfected cells. The mRNA expression of proinflammatory mediator *LITAF*, Treg cytokine (*TGF-β4*), toll-like receptor (*TLR21*) and *TNFSF13B* was also significantly increased in HD11 cells (Figure 7A). These results indicate that chicken LILRA2 and LILRA6 induce the expression of various cytokines and toll-like receptors in HD11 cells, however LILRA6 is more active compared to that of LILRA2. 

Moreover, we measured Th1 (IFN-γ) and Th17 (IL-17A and IL-12p40) cytokine production at the protein level in HD11 cell supernatants after LILRA2, LILRA6 and mock control transfection by ELISA (Figure 7B). The results revealed that IL-12p40 protein expression was significantly higher after LILRA2 (126.80 ng/mL) and LILRA6 (233.95 ng/mL) transfection in HD11 cells than that of cells transfected with the mock control. The expression levels of IL-17A and IFN-γ were also increased by LILRA2 (30.61 and 39.94 ng/mL, respectively) and LILRA6 (42.45 and 46.25 ng/mL, respectively) in HD11 cells (Figure 7B). Collectively, these results suggest that LILRA2 and LILRA6 might promote cellular development and induce in vitro production of Th1/17 cytokines in activated chicken HD11 cells. 

## 3. Discussion

The LILRA genes, including LILRA1–6*,* play an important role in the immune system of humans [1,7]. In chicken, some genes of the Ig superfamily such as TREM, CD300, SIRP, chicken Ig-like receptors (CHIR)-A, CHIR-B, CHIR-AB homologs and chicken leukocyte immunoglobulin receptor (LIR) were identified using bioinformatics approaches that may be important role in immune responses to high and low avian influenza virus [4,19,20,21]. However, functional characterization of the activating group of these genes has not yet been carried out in avian species including chickens. To our knowledge, the present study is the first to undertake the isolation and functional characterization of two activating genes, LILRA2 and LILRA6, in chickens. Although LILRA2 and LILRA6 were identified more than a decade ago, their biological significance and mechanism are poorly understood in mammals. It is interesting to identify the biological differences in structure and mechanism between LILRA2 and LILRA6 in chickens. Chicken LILRA2 and LILRA6 are glycoproteins composed of 306- and 320-aa, respectively and they contain a putative signal peptide, Ig-like domain, transmembrane region and cytoplasmic domain, which are common structural characteristics of the activating group of proteins [7,23]. In contrast to the human homologue, chicken LILRA2 includes one Ig-like domain (D1) and LILRA6 includes two Ig-like domains (D1–D2), indicating functional differences between LILRA2 and LILRA6 [22]. The location of two disulfide bonds in the D1–D2 domains of human LILRA2 and LILRA6 shows that they are successfully refolded and related to the main structure of the C2 type Ig-like domain and the C2 type Ig-like domains within the human LILR family are referred to as D1–D4 domain [22]. Because the C2 type Ig-like domains fold in a characteristic way for each receptor and contain a distinct binding site, it is possible to group receptor domains on a functional basis and determine which Ig superfamily receptor group they belong to [10]. Conversely, the cytoplasmic domains of LILRA2 and LILRA6 in chickens (55- and 71-aa, respectively) are longer than that of homologous human and mouse domains (13-aa). The different length of the cytoplasmic domains in LILRA2 and LILRA6 may differ in their binding affinities and regulating immune responses in chicken and human. In addition, the transmembrane regions of chicken LILRA2 and LILRA6 have a conserved arginine residue (4^th^ and 7^th^ position) that is important for activating Ig-like receptors, which associate with ITAM-containing FcɛRIγ or heterodimers of FcɛRIγ and CD3ζ [9]. Moreover, activating signaling through ITAM-containing FcɛRIγ leads to an oxidative burst and cytokine release and phagocytosis by macrophages indicates a pivotal role for FcγRs in the efficient MHC class-I restricted presentation of antigens [28,29]. Therefore, these results suggest that chicken LILRA2 and LILRA6 genes may differ in the capacity of their ligand binding sites and regulation of immunity. Comparison of amino acid sequence identities and similarities, sequence alignment and construction of a phylogenetic tree of chicken LILRA2 and LILRA6 showed that the chicken LILRA2 and LILRA6 are highly conserved and closely related to those of mammalian species, indicates that the chicken LILRA2 and LILRA6 are the orthologues of mammalian proteins. Moreover, expression of *LILRA2* and *LILRA6* mRNA in normal tissues and resting cells of chickens were slightly different but were generally upregulated in the small intestine and HD11 macrophage cell line. There is a limit to discussion due to lack of research regarding the role of chicken LILRA2 and LILRA6 in response to pathogens. 

In humans, binding of activating groups to HLA-A, -B and -C and other non-classical class I molecules such as HLA-E, HLA-G and HLA-F, are evaluated on several cell types such as NK cells, monocytes, macrophages, DCs and granulocytes [7,23]. On the other hand, recent study demonstrated that all nucleated cells express MHC class I molecules when cells infected with pathogen such as Marek’s disease [30], chronic autoimmune disease [31]. In the present study, immunoprecipitation, FACS and qRT-PCR analyses of LILRA2 and LILRA6 transfected HD11 cells compared with mock control suggest that LILRA6 strongly bound to MHC class I and β2m but LILRA2 did not. Moreover, LILRA6 significantly upregulated MHC class I and pathway genes such as *TAP1* and *TAP2* that are essential for antigen presentation but LILRA2 downregulated *TAP1* and *TAP2*. The findings that MHC class I is strongly recognized by LILRA6 but not LILRA2, in macrophages indicates that LILRA6 is an important activating receptor, playing an essential role in immune regulation and is capable of fine tuning innate immune responses in macrophages (Figure 3). Future analyses regarding the in vivo function of LILRA2 and LILRA6 are required to reveal if these two molecules play redundant or more exclusive roles in MHC class I-dependent immune responses.

Mammalian LILRAs contain a short cytoplasmic tail and a highly charged transmembrane domain that associates with the FcRγ. The binding of FcRs to the Fc region of immunoglobulins induces the activation of Src family kinases, which phosphorylate tyrosine residues within the ITAM motifs associated with FcRγ, activating the downstream signaling pathways [32,33]. The tyrosine phosphatase of Src family members and SHP2 associate with phosphorylated LILRAs in human in B-cells, macrophages and NK cells and activate the signaling pathways [3,7,33]. In our study, the level of Src kinases and SHP2 activation is significantly higher in LILRA6 than that of LILRA2 transfected HD11 cells. Moreover, our results revealed that the protein expression levels of Src family members were higher than SHP2 in LILRA6 transfected HD11 cell (Figure 4C). These results indicate that LILRA6 may be activated and induce more members of Src family such as SHP1, SH2-containing inositol phosphatase (SHIP) and Src homology 3 (SH3) [34,35,36]. Recent studies suggest that Src and SHP2 are involved in the signaling pathways of a variety of growth factors and cytokines such as JAK-STAT and MAPK signaling pathways, play an important role in transducing signal relay from the cell surface to the nucleus and are a critical intracellular regulator in mediating cell proliferation and differentiation [32,33,37]. Collectively, these results indicate that chicken LILRA2 and LILRA6 may differ in the regulation of signaling pathways and cytokine production. 

Moreover, the JAK-STAT signaling pathway plays an essential role in the activation of transcription factors that induce cytokines and cell differentiation [38]. In recent studies, it has been shown that LILRA2 and LILRA6 activate cytokine expression in macrophages [3,6] but the mechanism by which this occurs is not clear. On the other hand, SHP2 and Src tyrosine phosphatases are involved in the JAK-STAT signaling pathway [32,33,37]. A deeper understanding of the intracellular signal transduction pathways initiated by LILRA2 or LILRA6 is required to understand how they activate immune-related gene expression. Moreover, LILRA genes (LILRA1, 3 and 5) has been shown to play an important role in innate immune responses through regulation of the ERK/MEK [15], TLR [3,39] and JNK/p38MAPK [16] signaling pathways, as well as the antigen-presenting phenotype and cytokine production [3,6,9]. Increased expression levels of phosphorylated STAT1, STAT3 and JAK2 and un-phosphorylated STAT1/3, JAK2 and TYK2 molecules in response to LILRA2 and LILRA6 transfected in HD11 cells were detected by western blot and FACS analyses. A significant upregulation of *STAT1/3*, *JAK2* and *TYK2* mRNAs was detected by qRT-PCR. These results indicate that phosphorylated Src and SHP2 protein and mRNA expression were significantly increased, similar to that of p-JAK2 and p-STAT1/3. It is understood that p-JAK2 and p-STAT1/3 are recruited by the SH2 domain of the p-SHP2 protein and Src kinase [38]. Our results indicate that LILRA2 and LILRA6 activate and regulate the JAK/STAT signaling pathway to control the immune system. 

Src family tyrosine kinases are typical TCR signaling molecules that can activate NF-κB, PI3K/AKT and Ras/MAPK signaling pathways [37]. The NF-κB and AKT/MAPK signaling pathways are central regulators of innate and adaptive immune responses [40]. Recently, activating groups of LILR genes (LILRA1, 3, 4 and 5) were shown to induce ERK1/2 MAPK and Ras/AKT signaling pathways in humans [3,5,7,11,14]; however, the role of NF-κB, PI3K/AKT and ERK1/2 MAPK signaling pathways induced by chicken LILRA2 and LILRA6 is not clear. In this study, we first demonstrated that LILRA2 and LILRA6 transfected in HD11 cells activate phosphorylation of key regulators of NF-κB, PI3K/AKT and ERK1/2 MAPK signaling pathways. Chicken LILRA2 and LILRA6 induced the expression of phosphorylated NF-κB1, TAK1, AKT1 and ERK1/2 as detected by western blotting, FACS and qRT-PCR analyses. Previous reports demonstrated that the activation and interaction of STAT1 and NF-κB1 play a key role in regulating gene promoters, activate innate and adaptive immune responses and also enhance the production of Th1 and Th17 cytokines [41] as well as chemokines and toll-like receptors [42]. In addition, STAT3/NF-κB1 signaling pathways play important roles in promoting the development and progression of several cancers and also in controlling the immune response [43]. Thus, our data showed that chicken LILRA2 and LILRA6 induced the JAK-STAT and NF-κB signaling pathways and promoted the interaction/communication between phosphorylated STAT1/3 and NF-κB1, thereby controlling the expression of cytokines and the resulting immune responses. TAK1 is a mitogen-activated protein kinase kinase kinase (MAPKKK), which is activated by proinflammatory signaling and the toll-like receptor family [44,45]. TAK1 functions with TGF-β activated kinase (TAB) family genes to activate downstream kinases, leading to activation of NF-κB1 and MAPK signaling pathways [44,45]. Previous studies have indicated that cytokines such as IL-17A and IL-6 induce phosphorylated TAK1, STAT3, JAK2 and NF-κB1 and promote the association between TAK1 and STAT3 as well as JAK2 and NF-κB1 which regulate cytokine production [44,45]. Moreover, the interaction of major pathways including JAK-STAT, ERK1/2 MAPK and PI3K/AKT in the regulation of development, proliferation and differentiation of multiple cell types, particularly immune cells and hematopoietic cells, has been reported [44,45]. Therefore, our results suggest that LILRA2 and LILRA6 activate JAK-STAT, NF-κB, ERK1/2 MAPK and PI3K/AKT signaling pathways and interaction between them induces or controls cytokine production in HD11 cells. A previous study reported that IL-17A and IFN-γ are produced from Th1 and Th17 cells and induce the production of other cytokines such as IL-1β, IL-6, TNF-α and TGFβ-4 in human cells [46]. We showed that chicken LILRA2 and LILRA6 induce Th1, Th2 and Th17 cytokines, so they may act as immunomodulatory activators of the JAK-STAT, NF-κB, PI3K/Akt and MAPK signaling pathways.

In conclusion, this is the first report on the cloning, structural and functional analysis of the novel LILRA2 and LILRA6 in chickens. We showed that LILRA6 binds to MHC class I, β2m and other molecules involved in class I antigen presentation, processing and regulation of immune responses. Both LILRA2 and LILRA6 activate the phosphorylation of Src kinases and SHP2 that are modulators for signaling pathways in HD11 cells. Specifically, LILRA2 and LILRA6 induced and regulated the JAK-STAT, NF-κB, PI3K/AKT and ERK1/2 MAPK signaling pathways and upregulated Th1, Th2 and Th17 cytokines and toll-like receptor in HD11 cells. Collectively, both LILRA2 and LILRA6 are essential for macrophage mediated immune responses and they have the potential to complement the innate and adaptive immune system against pathogens.

## 4. Materials and Methods

### 4.1. Reagents and Antibodies

Mouse monoclonal GFPuv/eGFP antibody was purchased from R&D Systems (Minneapolis, MN, USA). Mouse anti-chicken MHC class I-PE and mouse anti-chicken β2-microglobulin (β2m)-PE antibodies were purchased from Southern Biotech (Birmingham, AL, USA). The following reagents/antibodies were either purchased from, or provided by, each respective company: rabbit anti-chicken STAT1 (phospho-Ser^727^), anti-chicken STAT3 (phospho-Ser^727^) and anti-chicken JAK2 (phospho-Tyr^1007^/Tyr^1008^) (Santa Cruz Biotech, Dallas, TX, USA); anti-chicken STAT1, anti-chicken STAT3 antibodies, horseradish peroxidase (HRP)-linked anti-rabbit secondary antibodies and Protein G–Sepharose beads (Sigma-Aldrich, St. Louis, MO, USA); anti-chicken SHP2 (phospho-Tyr^542^), anti-chicken NF-κB1 (phospho-Ser^933^, p50/100), anti-chicken TAK1 (phospho-Ser^192^), anti-chicken JAK2 and anti-chicken TYK2 antibodies (Biorbyt, San Francisco, CA, USA); rabbit anti-chicken GAPDH antibody (Abcam, Cambridge, MA, USA); Alexa Fluor 488 goat anti-rabbit IgG (H + L) secondary antibody (Invitrogen); biotin goat anti-mouse IgG (H + L) secondary antibody (BD Biosciences, San Jose, CA, USA); rabbit anti-chicken p44/42 MAPK (ERK1/2) (phospho-Thr^202^/Tyr^204^), anti-chicken Src family (phospho-Tyr^416^) and anti-chicken AKT (phospho-Ser^473^) antibodies (Cell Signaling, Danvers, MA, USA); goat anti-chicken IgG-PE antibody (EMD Milipore, Burlington, MA, USA); monoclonal anti-chicken IL-12p40 antibody (Kingfisher Biotech, Saint Paul, MN, USA); monoclonal anti-chicken IFN-γ and IL-17A antibodies (kindly provided by Dr. Hyun S. Lillehoj, USDA); and EZ-Link Sulfo-NHS-LC-Biotin, goat anti-mouse IgG secondary antibody linked HRP conjugate, rabbit anti-chicken IgG and HRP-conjugated streptavidin (Thermo Scientific, Waltham, MA, USA). 

### 4.2. Cloning and Sequencing Analysis of Chicken LILRA2 and LILRA6

To clone full-length chicken *LILRA2* and *LILRA6*, the predicted *LILRA2* and *LILRA6* coding sequences (CDS) (GenBank accession no. XM_004949812.1 and XM_003643874.2, respectively) were amplified with the total RNA from chicken intestinal tissue. To verify the sequences, primers were designed using the Lasergene software (DNASTAR Inc. Madison, WI, USA) and were synthesized by Genotech Co. Ltd. (Daejeon, South Korea); LILRA2 F: GCG GCC GCA TGA AAG GGG AAG CGG ATC; LILRA2 R: CCT CTA GAG CGC GGT AAA TCA GTG CT; LILRA6 F: GCG GCC GCA TGG TAT CAA TGG TGG TGG C; LILRA6 R: CCT CTA GAG GGG TCC CTG ACC CAA A. *LILRA2* and *LILRA6* were cloned into the pCR2.1-TOPO vector (Invitrogen) as described previously by Truong et al. [47,48]. Protein identification was conducted using the Expert Protein Analysis System (ExPASy; http://www.expasy.org/tools/) and multiple sequence alignment was performed using the Lasergene software. Phylogenetic analyses of amino acid sequences of LILRA2 and LILRA6 groups were constructed using the neighbor-joining method with a bootstrap value of 1000 in the MEGA6 program [49]. Signal peptides were predicted using the SignalP v.4.1 software [50] and glycosylation motifs were predicted using the NetOGlyc v.4.0 software [51]. The Ig domains, a transmembrane domain and cytoplasmic region were predicted using the InterPro v.56.0 software [52].

### 4.3. Chicken Tissues and Cell Culture

ROSS 308 broiler chicks purchased from YangJi Hatchery, Pyeongtaek, Republic of Korea were given unlimited access to antibiotic-free feed and water. A total of 17 tissue samples were collected from 14-days-old chicks and the samples were placed in liquid nitrogen for total RNA extraction. CD4^+^ and CD8^+^ T cells were isolated from the spleens of chicks as described previously by Truong et al. [47,48]. The chicken CU91 T [53], macrophage (HD11) [54], DT40 B [55], fibroblast (OU2) [56] and RP9 B (LSCC-RP9) [57] cell lines were grown in Dulbecco’s modified Eagle medium (DMEM; Invitrogen, Carlsbad, CA, USA) containing 100 IU/mL penicillin, 100 mg/mL streptomycin and 10% heat-inactivated fetal bovine serum (FBS; Invitrogen) in a humidified 5% CO_2_ atmosphere at 41 °C. All animal experiments were reviewed and approved by the Institutional Animal Care and Use Committee at Chung-Ang University (201600108), Anseong, Republic of Korea.

### 4.4. Vector Construction and Cell Transfection

Full-length CDS of *LILRA2* and *LILRA6*, originally cloned into pCR2.1, were excised using *NotI/Xbal* (Bioneer Corp, Daejeon, South Korea) and cloned into a eukaryotic expression vector pcDNA3-eGFP (Addgene, Cambridge, MA, USA), followed by transformation into *E. coli* BL21 (Invitrogen) and the positive clones were sequenced at Genotech. The plasmids were extracted and endotoxins were removed using the PureYield Plasmid Midiprep System (Promega, Madison, WI, USA) as per the manufacturer’s instructions. Chicken HD11 cells were transiently transfected with a pcDNA3-eGFP vector, containing the CDS of either LILRA2 or LILRA6 using Lipofectamine 3000 transfection reagent (Invitrogen), following the manufacturer’s protocol. A mock transfection was also performed using the empty pcDNA3-eGFP vector. A total of 1.0 × 10^6^ cells was transfected with 4 µg of plasmid in 6-well plates and transfected cells were immediately transferred on ice after 72 h and subsequently pelleted by centrifugation for further analysis. 

### 4.5. Cytotoxicity Test

To determine the cytotoxicity of LILRA2 and LILRA6 linked pcDNA3-eGFP vector and empty vector transfected into HD11 cells after 72 h cell proliferation and nitric oxide (NO) production assays were performed in 96-well plates according to well-established protocols [58]. The nitrite content and cell proliferation were measured using the Griess reagent (Sigma-Aldrich) and Cell Counting Kit-8 (Dojindo Molecular Technologies, Inc., Mashikimachi, Kumamoto, Japan), respectively. Lipofectamine 3000 reagent and DMEM medium were used as controls.

### 4.6. Flow Cytometry

Cells were re-suspended in staining buffer (10% FBS, 15 mM HEPES and 2 mM EDTA in PBS), primary antibodies were added to 1.0 × 10^6^ cells and the cells were incubated for 30 min on ice. To assess expression of LILRA2/6-eGFP binding with MHC class I and β2m, anti-MHC class I-PE and anti- β2m-PE antibodies were applied, followed by a single wash with staining buffer. Next, to investigate how LILRA2- and LILRA6-eGFP activate and regulate other signaling pathways, primary antibodies (pSRC, pSHP2, pSTAT1, pSTAT3, pJAK2, pERK1/2, pNF-κB1, pTAK1 and pAkt1) were added and incubated for 30 min on ice, followed by Alexa Fluor 488-conjugated anti-rabbit secondary antibody (Invitrogen) was added and incubated for 30 min on ice. Control sections were treated with goat anti-chicken IgG-PE or rabbit IgG only (unpublish data. Analysis by flow cytometry was performed with a BD FACSAria II cell sorter (BD Biosciences). Data were acquired using BD FACSDiva Version 6.1.3 and were analyzed using FlowJo 7.6.1 software. 

### 4.7. Immunoprecipitation and Western Blotting

Transfected cells were washed twice with ice-cold PBS and harvested in ice-cold RIPA buffer [50 mM Tris-HCl pH 7.5, 150 mM NaCl, 2 mM EDTA, 100 mM sodium fluoride, 0.1% (*w*/*v*) SDS, 0.5% (*w*/*v*) sodium deoxycholate, 1% Triton X-100, 10 mM sodium pyrophosphate and 10 mM sodium orthovanadate] containing complete EDTA-free protease inhibitor cocktail (Thermo Scientific). Cells were gently lysed for 30 min at 4 °C and centrifuged at 13,000× *g* for 15 min at 4 °C. Protein concentration was determined using the Coomassie (Bradford) protein assay kit (Thermo Scientific) in microplates according to the manufacturer’s instructions. The total protein (100 μg) was incubated with anti-eGFP mAb or anti-chicken GAPDH antibody at 4 °C overnight and then with 50 μL of Protein G–Sepharose beads for 2 h. Glyceraldehyde-3-phosphate dehydrogenase (GAPDH) is one of the key enzymes involved in glycolysis and is constitutively expressed in almost all tissues with high amounts. The immunoprecipitates were then washed thrice in RIPA buffer and solubilized in 2 × SDS-PAGE sample buffer. Samples were electrophoresed on Tris-glycine SDS-PAGE gels and transferred to polyvinylidene fluoride (PVDF) membranes (GE Healthcare, Marlborough, MA, USA). Membranes were blocked with 5% skim milk (Thermo Scientific) or 3% bovine serum albumin (BSA, Sigma-Aldrich) in PBS pH 7.4 containing 0.05% TWEEN 20 (PBST). Membranes were washed with PBST and incubated with anti-MHC class I and anti-β2m antibodies or signaling primary antibodies for overnight at 4 °C and the relevant secondary antibody in 2% non-fat milk or 0.5% BSA in PBST for 2 h at room temperature. Subsequently, membranes were developed using Western Lightning ECL Plus (Thermo Scientific) on Hyperfilm (GE Healthcare).

### 4.8. Enzyme-Linked Immunosorbent Assay (ELISA)

A 96-well plate (Nunc MaxiSorp, Nunc, Wiesbaden, Germany) was coated with 1:500 dilutions of monoclonal IFN-γ, IL-12p40, or IL-17A antibodies for 7 days at 4 °C, as previously described [58]. After blocking with 5% skim milk for 1 h at room temperature, the plate was incubated with culture supernatants of transfected cells, or different dilutions of recombinant IFN-γ, IL-12p40, or IL-17A, overnight at 4 °C. Following incubation with biotinylated-IFN-γ, -IL-12p40, or -IL-17A antibodies, HRP-conjugated streptavidin was added and incubated for 2 h at room temperature. The substrate 3,3′,5,5′-Tetramethylbenzidine (TMB, Thermo Scientific) was used as a chemiluminescent substrate and luminescence was measured in a Hybrid Microplate Reader (Epoch, BioTek Instruments, Winooski, VT, USA) at 450 nm. The protein of IFN-γ, IL-12p40, or IL-17A level were calculated based on a standard curve constructed with IFN-γ, IL-12p40, or IL-17A antibodies, respectively.

### 4.9. RNA Extraction and cDNA Synthesis

After transfection for 72 h with a pcDNA3-eGFP vector containing the CDS of LILRA2, LILRA6 and mock control, cells were washed with ice-cold PBS and total RNA was extracted using TRIzol (Invitrogen). RNA was diluted with 20 µL of RNase-free H_2_O and the concentration was determined using the Hybrid Microplate Reader. For cDNA synthesis, up to 2 µg of RNA was treated with 1.0 unit of DNase I and 1.0 μL of 10× reaction buffer (Thermo Scientific), then incubated for 30 min at 37 °C. Subsequently, 1.0 μL of 50 mM EDTA was added and heated to 65 °C for 10 min to inactivate the DNase I, then reverse transcribed using the Maxima First Strand cDNA Synthesis Kit (Thermo Scientific), according to the manufacturer’s recommendations.

### 4.10. Quantitative Reverse Transcription PCR (qRT-PCR)

To analyze the expression of cytokines, we designed primers using Lasergene software (Table S1) and performed qRT-PCR using 2× Power SYBR Green Master Mix (Roche, Indianapolis, IN, USA), with the LightCycler 96 system (Roche). Chicken *GAPDH* was used as an internal control gene to normalize cytokine expression. The relative quantification of gene-specific expression was calculated using the 2^−ΔΔ*C*t^ method after normalization to *GAPDH* [59]. All qRT-PCR was performed in triplicate.

### 4.11. Statistical Analysis

Measurement data are presented as the mean ± SEM of at least 3 replicates. Statistical analysis was performed using IBM SPSS software (SPSS 23.0 for Windows; IBM, Chicago, IL, USA). A *p*-value < 0.05 was considered to be statistically significant. Differences among the groups (treatment and control) were tested by the Duncan’s multiple comparison method. 

## Figures and Tables

**Table 1:** Similarities (upper) and identities (below) of chicken LILRA2 and LILRA6 to homologues of other species.

Genes	Species	LILRA2	LILRA6	LILRA2	LILRA6	LILRA2	LILRA6	LILRA2	LILRA6	LILRA2	LILRA6	GenBank Acc
		Chicken		Human		Monkey		Chimpanzee		Pig		
LILRA2	Chicken		50.08	38.97	39.50	27.86	39.15	38.97	38.97	55.37	39.15	XP_004949869
LILRA6		66.13		47.08	48.32	36.68	46.20	46.56	47.44	59.78	46.91	XP_003643922
LILRA2	Human	20.81	28.57		83.77	78.83	78.13	98.58	83.42	59.25	78.48	NP_001124389
LILRA6		20.45	28.57	72.83		71.25	83.77	83.24	94.53	60.14	82.36	NP_077294
LILRA2	Monkey	19.70	17.81	70.37	58.02		68.95	78.13	70.54	46.03	65.25	NP_001035761
LILRA6		20.98	27.33	67.54	75.66	55.55		78.13	83.95	56.26	76.54	NP_001035764
LILRA2	Chimpanzee	20.45	28.74	96.64	73.01	70.37	67.54		83.24	59.08	77.95	XP_009434592
LILRA6		20.81	28.39	73.19	90.82	56.96	76.89	73.36		61.19	81.83	NP_001009056
LILRA2	Pig	37.56	44.26	45.50	46.73	31.04	43.03	45.14	47.26		64.02	XP_013846133
LILRA6		20.63	26.63	63.49	69.31	50.08	63.49	64.02	69.48	49.91		XP_003134221

## Supplementary Materials

Supplementary materials can be found at http://www.mdpi.com/1422-0067/19/9/2710/s1.

## Abbreviations

NKs	natural killer cells
JAK	Janus kinase
STAT	signal transducer and activator of transcription
IL	interleukin
TNF	tumor necrosis factor
RT	reverse transcription
TYK2	tyrosine kinase 2
TGFβ	transforming growth factor beta
SH2	Src homology 2-containing tyrosine phosphatase 2
NF-κB	nuclear factor kappa B
TAK1	mitogen-activated protein kinase kinase kinase 7
MAPK	mitogen-activated protein kinases
Erk	Extracellular Signal-Regulated Kinase
PI3K	Phosphoinositide-3-Kinase
JNK	C-Jun N-Terminal Kinase
LILR	leukocyte immunoglobulin-like receptor
ITAMs	immunoreceptor tyrosine-based activation motifs
FcRγ	Fc receptor γ
