# Supplementary material for: Leukocyte Immunoglobulin-Like Receptors A2 and A6 are Expressed in Avian Macrophages and Modulate Cytokine Production by Activating Multiple Signaling Pathways
Source: Int J Mol Sci. 2018 Sep 11;19(9):2710. doi: 10.3390/ijms19092710 (PMC6163679; doi:10.3390/ijms19092710)
Supplement: Supplementary file 1 [file ijms-19-02710-s001.zip › LILRA2-6-Supp-Table S1-20180808.docx]

**Table S1**. Primer sequences for the gene expression analysis by quantitative real-time PCR.

| Primer | F/R | Nucleotide sequence (5′-3′) | Accession No |
| --- | --- | --- | --- |
| GAPDH | F | TGCTGCCCAGAACATCATCC | NM_204305 |
|  | R | ACGGCAGGTCAGGTCAACAA |  |
| STAT1 | F | TTGTAACTTCGCTATTGGTATTCC | NM_001012914 |
|  | R | TTCCGTGATGTGTCTTCCTTC |  |
| STAT3 | F | AGGGCCAGGTGTGAACTACT | NM_001030931 |
|  | R | CCAGCCAGACCCAGAAAG |  |
| JAK2 | F | CAGATTTCAGGCCGTCATTT | NM_001030538 |
|  | R | ATCCAAGAGCTCCAGTTCGTAT |  |
| TYK2 | F | GCCCCATGCAGGAGGAAT | XM_427671 |
|  | R | CTTTGCCACAGCCAGAATCAC |  |
| SHP2 | F | ATGTTGGTGGAGGGGAGAA | NM_204968 |
|  | R | GGGGCTGCTTGAGTTGC |  |
| TAK1 | F | CCAGGAAACGGACAGCAGAG | XM_015284677 |
|  | R | GGTTGGTCCCGAGGTAGTGA |  |
| NF-κB1 | F | AGAAAAGCTGGGTCTTGGCA | NM_205134 |
|  | R | CCATCTGTGTCAAAGCAGCG |  |
| IFN-γ | F | AGCTGACGACGGTGGACCTATTATT | HQ739082 |
|  | R | GGCTTTGCGCTGGATTC |  |
| IL-6 | F | CAAGGTGACGGAGGAGGAC | JQ897539 |
|  | R | TGGCGAGGAGGGATTTCT |  |
| IL-17A | F | TGTCTCCGATCCCTTGTTCT | AM773756 |
|  | R | GTCCTGGCCGTATCACCTT |  |
| IL-17F | F | CTCCGATCCCTTATTCTCCTC | NM_204460 |
|  | R | GTCCTGGCCGTATCACCTT |  |
| LITAF | F | TGTGTATGTGCAGCAACCCGTAGT | AY765397 |
|  | R | GGCATTGCAATTTGGACAGAAGT |  |
| TGF-β4 | F | CGTGCCCGTACATCTGGAG | JQ423909 |
|  | R | GAGGGGGTCGAGGGTCTG |  |
| IL-1β | F | TCGGGTTGGTTGGTGATG | NM_204524 |
|  | R | TGGGCATCAAGGGCTACA |  |
| IL-12p40 | F | AGATGCTGGCAACTACACCTG | NM_213571 |
|  | R | CATTTGCCCATTGGAGTCTAC |  |
| IFN-ß | F | CTTGCCCACAACAAGACGTG | NM_001024836 |
|  | R | GTGTTTTGGAGTGTGTGGGC |  |
| IL-4 | F | AGCACTGCCACAAGAACCTG | NM_001007079 |
|  | R | CCTGCTGCCGTGGGACAT |  |
| IL-10 | F | CTGTCACCGCTTCTTCACCT | AJ621254 |
|  | R | ACTCCCCCATGGCTTTGTA |  |
| IL16 | F | TGCCTCACAAGAATCAACAACT | AB104417 |
|  | R | ATAGAGCCCTTCCCACCTTC |  |
| TLR21 | F | GCTCACAGGCAAAATCACG | XM_003641158 |
|  | R | AGCAGCAGGGTTCTCAGGT |  |
| ERK1 | F | GCAAGCTTTAGCCCATCCA | NM_204150 |
|  | R | GTCATCCAATTCCATATCAAACTT |  |
| ERK2 | F | CATCGCGACCTCAAACCTTC | AY033635 |
|  | R | TCCGGATCTGCAACACGAG |  |
| TNFSF13B | F | GTGCCTCTGTTTCTTCCTTCCT | NM_204327 |
|  | R | TCTCCCCCTGTTTCTGTTCCT |  |
| β2m | F | CAGGTGTACTCCCGCTTCC | NM_001001750 |
|  | R | GGCACGCCATCCTTCAT |  |
| TAP1 | F | GGCGGCTGCACACTACC | AJ843261 |
|  | R | CCAGGGCTGAGAAACCACT |  |
| TAP2 | F | GACACCCGGCACCAGAT | NM_001099357 |
|  | R | CCTCCTCGCCATTGAAGA |  |
| TAK1 | F | CCAGGAAACGGACAGCAGAG | XM_015284677 |
|  | R | GGTTGGTCCCGAGGTAGTGA |  |
| BF-I | F | GCACAGCCCCATCCTCT | AM279341 |
|  | R | TGGCCCATCATTTTATTTCA |  |
| BF-IV | F | CCATCCGGGGGTATTATCA | AF013492 |
|  | R | TGGTGGGAACTGCCTCTG |  |
| MICA | F | GGGACTTCCTCGCCTTTGA | XM_003643799.2 |
|  | R | ATCCCTTCCTCCTCCCATCTC |  |
| LILRA2 | F | CTCCCCAAGTGTCTCATTTCTG | XM_004949812.1 |
|  | R | ATTTGGGGAGGTTATCTGGTTT |  |
| LILRA6 | F | GACACGACCGAGTTCTTCTTTG | XM_003643874.2 |
|  | R | CTCCACGGGGTCACTCTTCT |  |
