# Supplementary figures and images for: Leukocyte Immunoglobulin-Like Receptors A2 and A6 are Expressed in Avian Macrophages and Modulate Cytokine Production by Activating Multiple Signaling Pathways
Source: Int J Mol Sci. 2018 Sep 11;19(9):2710. doi: 10.3390/ijms19092710 (PMC6163679; doi:10.3390/ijms19092710)

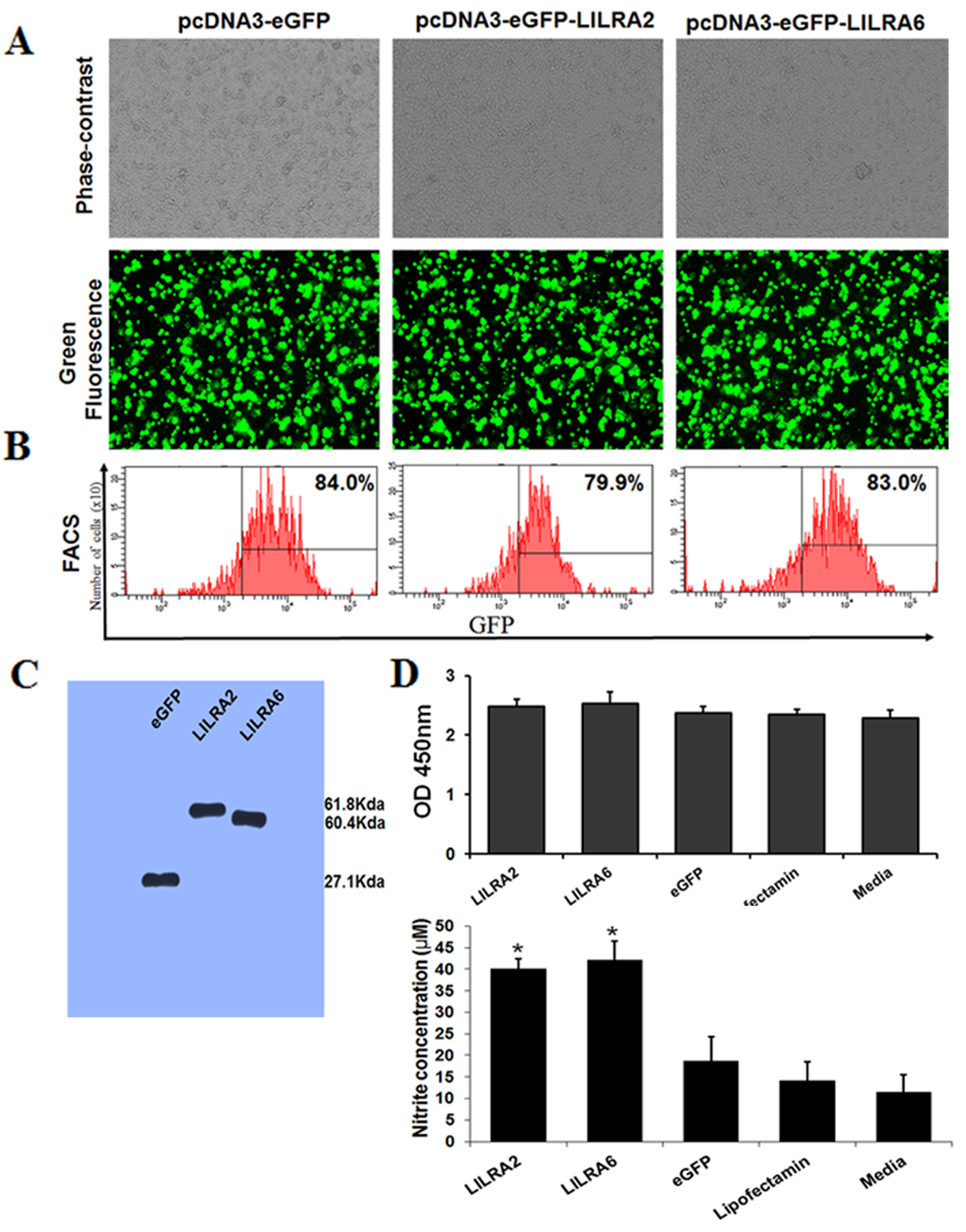

Supplement: Supplementary file 1 [file ijms-19-02710-s001.zip › Figure-S1-LILRA2-6-Transfection.jpg]
